# Vulvar Skin Disorders throughout Lifetime: About Some Representative Dermatoses

**DOI:** 10.1155/2014/595286

**Published:** 2014-01-08

**Authors:** Jean Doyen, Stéphanie Demoulin, Katty Delbecque, Frédéric Goffin, Frédéric Kridelka, Philippe Delvenne

**Affiliations:** ^1^Department of Gynecology and Obstetrics, Regional Hospital Citadelle, 4000 Liege, Belgium; ^2^Laboratory of Experimental Pathology, GIGA-Cancer, University of Liege, 4000 Liege, Belgium; ^3^Department of Pathology, University Hospital of Liege, 4000 Liege, Belgium

## Abstract

The objective of this paper is to present general considerations which should be kept in mind by clinicians in charge of women with vulvar diseases. Four representative vulvar dermatoses are described. Lichen simplex chronicus is a pathological condition related to chemical and mechanical irritant agents. Detrimental effects of these irritants, in the presence of other dermatoses, have to be considered when therapeutic responses are unsatisfactory. Lichen sclerosus is the most common vulvar dermatosis in elderly. However, it should be kept in mind that it may be diagnosed at any age. Lichen planus, in spite of sharing a similar range of etiological factors with lichen sclerosus, is a very distinct entity. Finally, Paget's disease, although rare, is also described especially because of the challenge it represents both clinically and therapeutically.

## 1. Introduction

Vulva is, for many reasons, to be regarded as a particular anatomic area. Considering anatomy and patient's own point of view, this region is of course not easily self-observable and, as a part of genitalia, often quite unknown and mysterious, for cultural or emotional reasons. It is composed of several folds including clitoral hood, labia majora and minora, hymen, and anal margin. Microscopically, vulva is covered by different types of epithelia, depending on the area of interest, including, from its lateral to medial region, keratinized hair bearing skin, partially keratinized hairless skin, and, beyond Hart's line, mucous membrane of the vestibule. A large number and variety of adnexal structures are associated with vulvar skin in its different sections, such as pilosebaceous units, sebaceous and sweat glands, mucous secretory glands, muscle fibers, and deeper major or minor vestibular glands. Vicinity of underlying vascular structures can also modify vulvar aspects. Therefore, any component of blood and lymphatic vessels can be affected through malformations, tumors, or dystrophic changes.

From a pathological point of view, vulva, as a part of genitalia, can be affected by specific disorders such as multifocal HPV lesions of any degree or vulvar expression of a vaginal infection. Vulva can also exhibit specific dermatological diseases for which signs can be observed elsewhere on the body, such as in lichen sclerosus or psoriasis. However, vulva can also exhibit signs of a large variety of diseases, such as digestive, hematological, immunological, and endocrine disorders. This leads us to consider any vulvar disorder as a potential expression of a very large panel of diseases.

Clinically, if many vulvar lesions are reasonably characteristic, numerous clinical manifestations are not specific of one disorder and some diseases can express different morphological patterns. For these reasons, the revision should follow unexpected no-response to empirical treatment resulting from a clinical diagnosis. Frequently, pathological patterns cannot be automatically related to one single cause.

Biopsy is certainly an important diagnostic step in many circumstances. As biopsy is an invasive procedure, especially on the vulva, special care should be taken. Unless for very suspicious lesions that require prompt diagnosis, topical treatments, especially corticosteroids, should be stopped 3 to 4 weeks before performing biopsy to allow natural histological expression of the disease. Local anesthesia is mandatory and biopsy should be performed using a 4 to 5 mm punch device to avoid crushing artifacts occurring with biopsy forceps.

As a consequence of these considerations, it seems quite difficult to present a classification of disorders affecting vulva which all medical specialties would agree with. Classification of the Internal Society for the Study of Vulvar Diseases appears credible as it is periodically revisited and as it is the result of consensus between gynecologists, dermatologists, and pathologists. At the present time, 2006 ISSVD Classification [1] is still relevant. But, as this classification is of minor help for diagnosis, ISSVD formulated in 2011 a complementary classification as an approach to clinical diagnosis [2].

Clinicians dealing with vulvar complaints should always keep in mind these preliminary considerations and, as a consequence, be convinced that treating vulvar disorders needs a complete anamnestic investigation, examination of the lower genital tract, skin, and sometimes oral mucosae, and dialog between colleagues. Furthermore, special attention should be paid to psychosexual status of patients suffering from vulvar disorders, as they are often present, either as a cause or a consequence of the disease. Effect of vulvar diseases on self-regard, affective, and sexual life is potentially important. Therefore, time should be given for exhaustive explanations about etiology, nature, and course of the disease and consequences, if any, on sexual life. Causal treatment should also be associated with protective measures avoiding contacts with mechanical and chemical irritants. A nonexhaustive list of common recommendations includes avoiding fabric softeners, pads, detergents, cosmetic products containing color additives and flavors, and synthetic underwear. This is sometimes sufficient to eradicate irritant and contact dermatitis and limits risk of poor response to true dermatoses. In addition, any coexisting disorder, such as diabetes mellitus or urine incontinence, should be under control.

Too often, women suffering from vulvar complains, especially itching, are still nowadays offered symptomatic treatment without diagnostic process or even without examination, such as over-the-counter delivery of any cream. This may, in some cases, lead, even in young people, to delayed diagnosis of life-threatening diseases, such as squamous cell carcinoma (SCC). For this reason, in the presence of vulvar complains, a diagnostic pathway including systematic physical examination is always mandatory.

This raises the question of vulvar screening. Even if vulva shares some diseases with cervix and vagina, especially through HPV dependent lesions, the place of screening is not comparable. Indeed, vulvar cancer is ten times less frequent than cervical one. Most vulvar lesions are symptomatic, and vulva is accessible to observation. As a consequence, vulvar cytology development has a minor place, if any. On the contrary, vulvoscopy has to be regarded as a valid tool, especially in complicated clinical presentations and in situations where there is a risk of intraepithelial neoplasia or microinvasion [3].

Of course, this paper does not pretend to overview all the aspects of vulvar diseases. Consequently, selecting some of the most representative vulvar dermatoses is subjective. The four selected vulvar diseases described below illustrate the variety of pathologies which may be encountered and for which awareness is necessary.

Lichen simplex chronicus, a nonspecific disorder, may be the expression of a so common contact dermatitis but may also reveal another underlying disease. Lichen sclerosus is the most current vulvar dermatosis in elderly and often easy to treat, requiring a sustained attention. Lichen planus, in spite of having some common points with the previous one, is clearly distinct and often more difficult to evaluate. Finally, vulvar Paget's disease remains a pathological condition difficult to handle because it is clinically nonspecific, rare, and easily missed and for which new treatments are necessary.

## 2. Lichen Simplex Chronicus

This disorder is a chronic eczematous condition characterized by intense pruritus. Rubbing and scratching produce poorly demarcated plaques of thickened lichenified skin. Scale may be subtle and results in slightly shining. Erosions and fissures can result from scratching and become infected.

In children, atopic dermatis can result in lichen simplex chronicus, most often characterized by a widespread inflammatory eruption instead of vulvar lesions. Adults are more likely to present genital eczema.

Treatment requires a three-steps process. The first step consists in identifying and eliminating all irritant and allergen exposures. Secondly, treatment requires the breaking of the itch-scratch-itch cycle, especially during nighttime sedation, with oral antihistamine drug taken at bedtime and high potency topical corticosteroid, once a day at bedtime. Additionally, identifying and treating concomitant infection are needed.

Clinical control after one month is recommended. In some cases, another underlying pruritic condition might be revealed. If treatment fails or lichen simplex chronicus recurs, attention should be paid to psychological status of the patient. Psychological disturbances may as well be a cause or a consequence of persisting or recurring lesions. They should be treated specifically with appropriate support. As a result, surgical resection of a lichenified prominent labia majora should be an exceptional procedure. Pathologic analysis is of course necessary.

## 3. Lichen Sclerosus

Lichen sclerosus is one of the most common dermatoses affecting vulva in elderly. The disease is sometimes asymptomatic, but pruritus is frequent and represents by far the main complaint. However, depending on the way disease has developed, other complaints may be expressed. Pain may result from fissures and induce, for example, dyspareunia or constipation when perianal. Dyspareunia may also be due to introitus narrowing.

**Figure 1:**
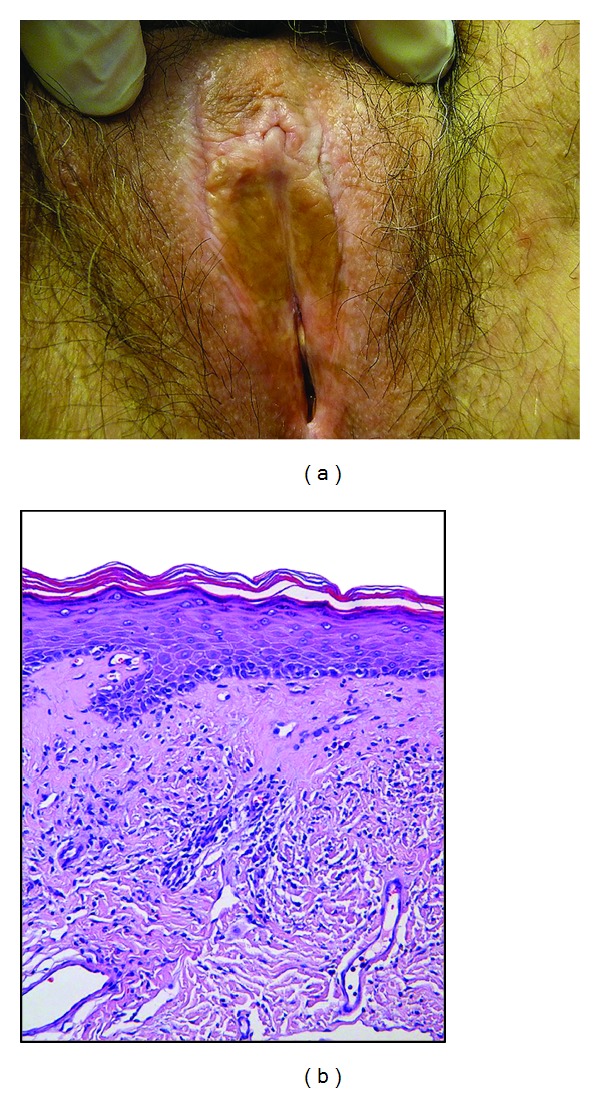
(a) Irregular white to brown patch on the vulvar skin of a 74-year-old woman. (b) Representative example of a lichen sclerosus biopsy specimen showing a thinned epidermis with slight hyperkeratosis, loss of the rete ridges, and a band of homogenized collagen below the dermoepidermal junction.

Pathology is characterized by epithelial thinning down, sclerosis of the upper derma, and inflammatory infiltrate underneath (Figure 1(b)). Hyperkeratotic changes may also occur in some locations. Thin epithelium was previously thought to represent an atrophic state but is probably due to accelerated maturation. For that reason, this disease is no more called lichen sclerosus atrophicus. As a consequence, androgen-containing creams are no more up to date treatments, as they are not justified and as they induce virilization, locally (clitoral hypertrophy), or, sometimes, with general signs.

Clinically, vulvar lichen sclerosus induces mainly whitening of the skin, due to sclerosis (Figure 1(a)). Topography is variable from focal to large bilateral extension and from periclitoral to perianal area. Labia minora are often affected, either thickened or reduced. Clitoral hood may merge, bury clitoris, and result in pseudocyst formation, containing smegma. Sclerosis of the introitus may result in sexual impairment and dyspareunia. Inflammatory stages during which pruritus may be especially present can induce some degree of stromal congestion and skin redness, fissuring, and hyperkeratotic reaction. Thinning of the skin, combined with itching, results in petechia. Dystrophy and chronic irritation are probably a favorable condition for inducing nuclear atypia in the deepest layers of epithelium, resulting in high grade vulvar intraepithelial neoplasia (VIN) of a differentiated type. As a consequence, most vulvar SCC of elderly occur on dystrophic vulva and result from degenerating focal VIN. Vagina is never affected. On the other hand, extragenital manifestations can be observed.

Etiology of lichen sclerosus is unknown but related to an autoimmune mechanism. Other autoimmune diseases occur in 21% of patients: thyroiditis (12%), alopecia areata (9%), and vitiligo (6%) are the most frequent ones. It is more frequent in women than in men. Genital localization can occur at all time from prepubertal age to elderly and is more common at these ages than in middle ages [4].

In children, lichen sclerosus may be difficult to diagnose in some instances, or diagnosis is simply not expected. This should explain why mean interval between symptoms and diagnosis is reported to be around 1.7 years. At this period of life, vulvar folds are not completely developed, and vulvar folds changes nearly do not appear. Whitening may be present. Fissures can be nearly the only manifestation, especially around anus, inducing pain and constipation. These children may be suspected to be victim of sexual abuse, and this should be carefully taken in consideration. Furthermore, lichen sclerosus and abuse may coexist, the second triggering the first one. For that reason, any unexplained observation such as poor response to treatment should raise special attention [4]. Any parasitic origin such as oxyurosis should also be considered.

In young women, clinical presentation is not especially different than in elderly, but special attention should be paid to long-term risk of atrophic changes due to their effect on sexual life.

In elderly, the most important part of the treatment is the control of pruritus. But sexual complains are not rare, because long-term progression of the disease, added to natural atrophic changes of lower genital tract, alters vestibular anatomy and function. Periodic examination of the vulva under treatment remains important at this age, even in the absence of complain, because any persistent dystrophic area should be suspected to bear epithelial atypia and represent a VIN focus.

Occurrence of SCC is debated in its causative relation to lichen sclerosus. Although this cancer occurs in no more than 5% of lichen sclerosus. In contrast, histologic signs of lichen sclerosus are present in 33% of cases of SCC of the vulva [4]. Older age and epithelial hyperplasia are independent risk factors. The presumed pathway would imply successively lichen sclerosus, hyperplasia, and lower atypia representing differentiated type VIN, then leading to carcinoma. However, this sequence is not well understood and even controversial [5].

Considering hypertrophic forms of lichen sclerosus as a potential precancerous disease is not unanimously admitted. In a short series of patients with hyperplastic changes in lichen sclerosus, in whom followup was available for at least 5 years, no progression to cancer was noticed [6]. This might be taken into consideration to avoid unuseful surgery. However, biopsy is necessary to assess the absence of atypia and observation is recommended in foci of hyperplastic epithelium in lichen sclerosus.

The presence of epithelial atypia in association with lichen sclerosus is also controversial. It is commonly admitted that VIN associated with lichen sclerosus is a differentiated type and that overexpression of p53 is present, contrary to what happens in undifferentiated type VIN, induced by HPV, and in which overexpression of p16 is encountered [5]. Once again, common link between differentiated type VIN and lichen sclerosus is debated [6], and at least one report previously showed that VIN associated with lichen sclerosus is most often of the undifferentiated type [7].

These data illustrate how it is difficult to obtain agreement between members from one or from different communities. This, in turn, affects dialog between clinicians and pathologists and sometimes makes surgical decisions difficult to discuss.

Treatment of lichen sclerosus is nearly always based on the use of potent corticosteroid cream. Application in the adequate area is to be teached, especially in elderly, because the area is not always easy to reach. Patients have to learn the way cream must be applied until complete skin absorption. Treatment effect has to be followed up on both complaints and objective vulvar appearance. This allows detecting wrong appliance of treatment or nonresponding suspect areas. Treatment must be adjusted, and applications reduced to minimal frequency and amount of cream needed. This reduces secondary effects risk of long-term use and leaves therapeutic margin if disease gets more active later. When disease is totally under control, stopping treatment for sometimes long periods should be debated. It is certainly indicated in younger patients and has to be discussed in older ones, who should always be followed up on a long-term basis, either treated or not, even if objective cancer occurrence risk is lower than 5%.

The place of topical calcineurin inhibitors in the treatment of lichen sclerosus and other conditions such as lichen planus is worth thinking about.

Topical calcineurin inhibitors are to be considered as a second-line treatment, when all reasonably possible adjustments of corticoid creams have been tested [8]. In some instances, corticoid creams are not or no more tolerated (delayed-type hypersensitivity reactions) or become less efficient, leading to increasing treatment doses (tachyphylaxis). They may also induce rebound erythroderma or lead to cutaneous atrophy. The main advantages of calcineurin inhibitors are to avoid tachyphylaxis and cutaneous atrophy. Nevertheless, they are associated with secondary effects. Topical calcineurin inhibitors commonly induce transient local burning or irritation. Facial flushing after alcohol ingestion and rosaceiform dermatitis may also less commonly occur [9].

Long-term safety profile of topical calcineurin inhibitors has been questioned, especially as they have been suspected to increase cancer risk. However, it seems reasonable to assess that this risk has been overestimated. It is, in fact, not higher than in the general population [10].

It has been demonstrated that potential systemic effects of topical calcineurin inhibitors are lower than that of corticosteroids, due to their weaker permeation potential. For that reason, lymphoproliferative changes observed in monkeys did not result in a higher risk in patients. Risk of immunosuppression was not documented by any increased risk of systemic or skin infections. Photocarcinogenicity was not described in patients using topical regimens [9]. Eventually, SCC occurring in the presence of topical calcineurin inhibitors was investigated by the US FDA. Twenty nine cases were reported worldwide and only 3 occurred in cases of lichen sclerosus [4].

Considering all these aspects, topical calcineurin inhibitors should be regarded as a second-line treatment. It has to be presented as an off-label option, if possible for short durations, owing to previous considerations and to their costs, clearly higher than that of corticosteroids [4].

Surgical treatment is limited to some indications: surgery of clitoral hood when clitoral phimosis is sexually detrimental or when a painful pseudocyst has developed; enlargement of introitus when superficial dyspareunia occurs; resection of dystrophic zones when biopsy demonstrates associated VIN or vulvectomy in the presence of carcinoma.

## 4. Lichen Planus

**Figure 2:**
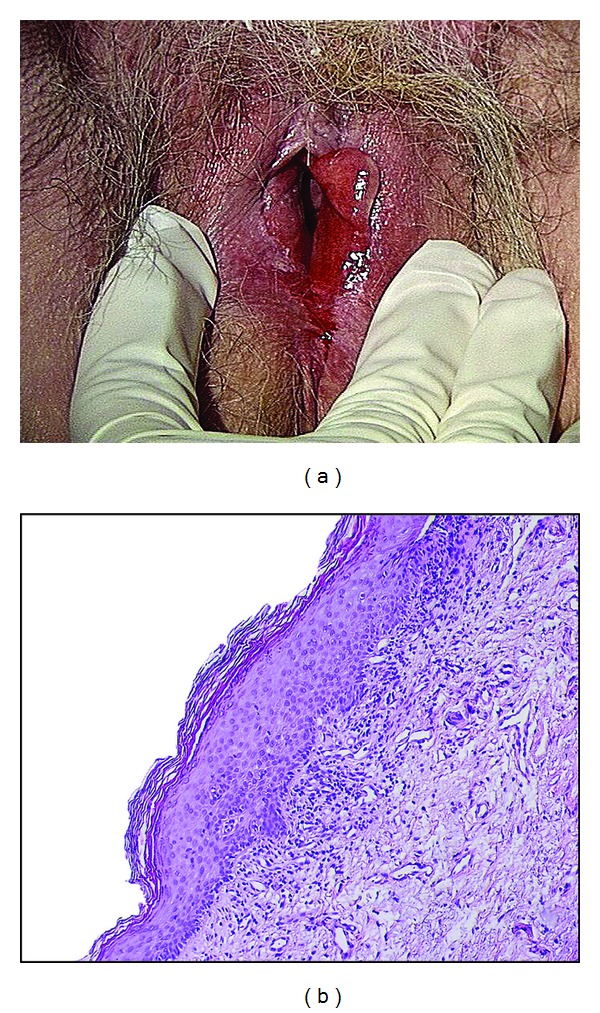
(a) Wide vestibular erythematous erosive lichen planus in a 79-year-old woman. (b) Representative example of a lichen planus biopsy specimen showing an irregular hyperkeratosis and a lymphoid dermal infiltrate appearing as a distinct band close to the epidermis.

Lichen planus (Figure 2) is a more uncommon condition that may be asymptomatic, itchy, or more frequently painful. This is mainly due to frequent erosive forms, especially those affecting vestibulae. Chronic vaginal discharge and dyspareunia are frequently associated.

Pathology may show different patterns, in relation to different clinical appearances. Epidermis can be thickened or erosive. Basal cell layer shows some areas of vacuolar degeneration. A band-like mononuclear infiltrate is present in the upper dermis (Figure 2(b)).

Clinically, vulvar lesions can be represented by different patterns, including violaceous shiny papules, reticular white networks, or erosive forms, especially on the vestibulae and vagina (Figure 2(a)). Actually, in contrast to lichen sclerosus, it can extend to vagina and turn, in this location, to extremely invalidating and difficult to treat erosive vaginitis. Extragenital and buccal locations are frequent. Erosive vulvovagino-gingival lichen planus is known as the syndrome of Hewitt and Pelisse.

Consensus on the diagnostic criteria for erosive vulvar lichen planus was recently defined by members of Vulvar Diseases Societies [11]. Nine criteria were gathered of which three should be present to support the diagnostic: (i) well-demarcated erosions/erythematous areas at the vaginal introitus; (ii) presence of a hyperkeratotic border to lesions and/or Wickham striae in surrounding skin; (iii) symptoms of pain/burning; (iv) scarring/loss of normal architecture; (v) presence of vaginal inflammation; (vi) involvement of other mucosal surfaces; (vii) presence of a well-defined inflammatory band involving the dermoepidermal junction; (viii) presence of an inflammatory band consisting predominantly of lymphocytes; and (ix) signs of basal layer degeneration.

Etiology is, as for lichen sclerosus, suspected to be autoimmune. Moreover, in some cases, mixed forms of lichen planus and sclerosus are encountered.

This disease is rare in younger and older patients. Most of affected people are middle aged around 30 to 60 years.

Treatment is targeted against complains. Pruritic lesions respond to local applications of highly potent corticoid creams, but response may be more difficult to achieve than in lichen sclerosus. Far more difficult is, anyway, the control of dyspareunia, related to vestibular and vaginal erosions. These conditions always respond to some degree to the same treatment, but results are often precarious, incomplete, or, in the end, not satisfactory. Topical corticoid use in the vagina may require specific preparations, incorporating, for example, corticoid drug in adeps solidus suppositories or in carbopol gel. Topical calcineurin inhibitors may have to be tried when results are not satisfactory. Systemic oral corticoids are considered when topical treatments fail.

Surgery is sometimes necessary to lyse vaginal adhesions or to restore vestibular anatomy in case of impaired sexual function. But these conditions are difficult to treat and satisfactory results are far from being the rule.

## 5. Vulvar Paget's Disease

Paget's disease is mainly a disease of elderly. Mean age is around 65 years of age. A special attention should be paid to this rare disease, because its clinical appearance might suggest other conditions, such as eczema, lichen simplex chronicus, psoriasis, high grade VIN, and others. This consideration illustrates the fact that a high degree of suspicion must legitimately lead to prompt biopsy in the presence of nonspecific clinical presentations, especially if a first-line treatment fails to induce clinical improvement. On the contrary, the nonspecific presentation and complaints, in frequently old patients, explain the time between onset of complaints and diagnosis, estimated around two years [12].

Here again, pruritus is a common initial symptom. Pain can substitute when erosion occurs. Complaints are limited to the often quite delineated involved area.

**Figure 3:**
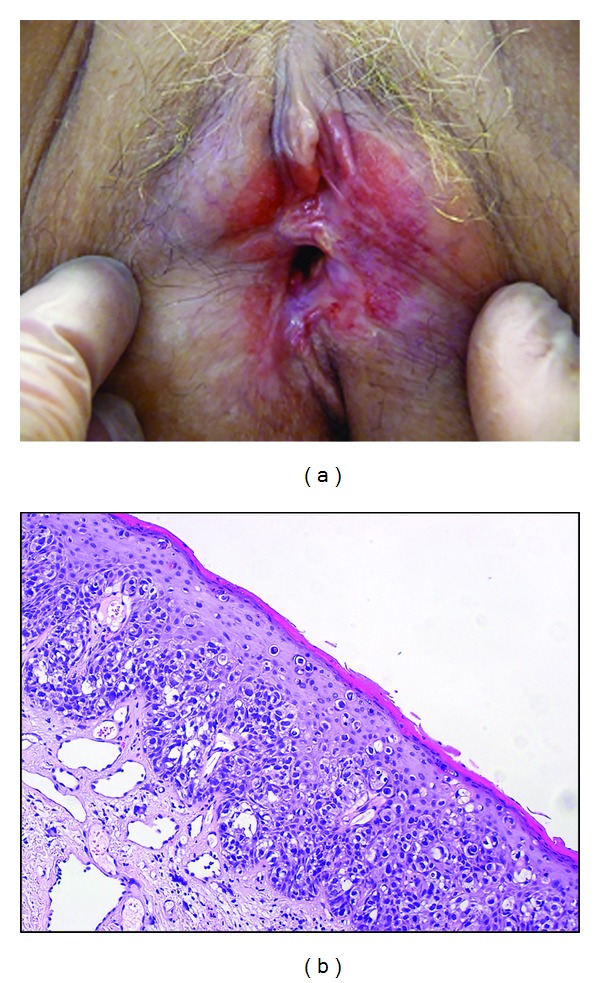
(a) Wide red area extending mainly on the left side of the vulva in a 65-year-old woman with Paget's disease. (b) Representative example of Paget's disease biopsy specimen showing many glandular neoplastic cells with clear cytoplasm present singly or in small nests within the epidermis.

Clinically, the lesion is often described as red, well-delineated, with a scaling or sometimes erosive surface, in the area of keratinized vulvar skin, and as far as perianal region (Figure 3(a)). The lesion is chronic, and a history of local discomfort may exist years before diagnosis. Early clinical signs are probably highly nonspecific, as it can be seen in early recurrences observed during followup, where still asymptomatic patients present minor erosions or slight focal redness.

Pathology is the only reliable key for diagnosis and requires scattered large clear muciparous cells with nuclear atypia, inside the epidermis (Figure 3(b)). They can be present also in skin appendages. Underlying chronic infiltrate is often described. Presence of these cells in the dermis reflects an invasive form, which is a rare occurrence of the disease.

Etiology is related to the glandular nature of abnormal cells and to the fact that Paget's disease, either mammary or extramammary, appears nearly exclusively in skin areas where apocrine glands are present. However, some believe that malignant cells are pluripotent epidermal ones.

In spite of the fact that invasive disease is rare, association with underlying internal malignancies is a well-known characteristic of the disease, but its frequency is diversely appreciated and should be probably around 10 to 20%. For that reason, different authors recommend routine endoscopic genital-urinary and intestinal tract exploration to be performed [13].

Treatment is mainly surgical, especially when diagnosed for the first time. Unfortunately, the procedure is far from being always satisfactory. The lesion is not well delineated as the clinical presentation might suggest, and the disease is often present, somewhat far from the apparent margin. Recommended surgical margin goes up to 2 to 3 centimeters outside apparent limit of the disease, depending on whether it seems well delineated or not. Some surgeons perform frozen section margins control during surgery, but even this precaution may lead to final pathology reporting up to 10% positive margins [13]. Others reported failure rates of 20 to 60%, even if variant surgical procedures, such as Mohs micrographic surgery, improve results with recurrence rates lowered down to 16% [12].

Other treatments are currently offered and include interferon alpha, topical fluorouracil, imiquimod, CO_2_ laser ablation, and, more recently, photodynamic therapy [14]. They should be considered as alternatives to surgery or to cure recurrences, while regarding radiotherapy as the ultimate solution, due to its long-term secondary effects, and taking into account the fact that any further treatment would possibly be difficult or impossible to use after radiotherapy.

Due to the nature of the disease, and its frequent microscopic extension to apparently normal surrounding skin, posttreatment biopsies are of low interest. Clinical remission and symptoms resolution are reasonable results for treatment [12].

## 6. Conclusion

The examples of these four frequent vulvar dermatoses underscore the importance of considering a wide range of diagnoses to obtain an accurate diagnosis. A close cooperation between clinicians and pathologists is also mandatory. Finally, exhaustive explanations relative to diagnosis and treatment and psychological support for the patient have to be integrated in the management of these diseases.
